# Inflammation-Driven Genomic Instability: A Pathway to Cancer Development and Therapy Resistance

**DOI:** 10.3390/ph18091406

**Published:** 2025-09-18

**Authors:** Nina Rembiałkowska, Zofia Kocik, Amelia Kłosińska, Maja Kübler, Agata Pałkiewicz, Weronika Rozmus, Mikołaj Sędzik, Helena Wojciechowska, Agnieszka Gajewska-Naryniecka

**Affiliations:** 1 Department of Molecular and Cellular Biology, Faculty of Pharmacy, Wroclaw Medical University, Borowska 211A, 50-556 Wroclaw, Poland; agnieszka.gajewska-naryniecka@umw.edu.pl; 2 Faculty of Medicine, Wroclaw Medical University, 50-367 Wroclaw, Poland; zofia.kocik@student.umw.edu.pl (Z.K.); amelia.klosinska@student.umw.edu.pl (A.K.); maja.kubler@student.umw.edu.pl (M.K.); agata.palkiewicz@student.umw.edu.pl (A.P.); weronika.rozmus@student.umw.edu.pl (W.R.); mikolaj.sedzik@student.umw.edu.pl (M.S.);

**Keywords:** chronic inflammation, DNA damage, cancer development, inflammatory signaling pathways, reactive oxygen and nitrogen species, cancer, immune response, cytokines

## Abstract

Chronic inflammation, while originally a protective physiological response, is increasingly recognized as a key contributor to carcinogenesis. Prolonged inflammatory signaling leads to the sustained production of reactive oxygen and nitrogen species (ROS/RNS), resulting in direct and indirect DNA damage, including base modifications, strand breaks, and DNA cross-linking. Simultaneously, pro-inflammatory mediators such as NF-κB, IL-6, and TNF-α can interfere with DNA repair mechanisms, altering the efficiency of key pathways such as base excision and mismatch repair. Immune cells infiltrating chronically inflamed tissues, including macrophages and neutrophils, further exacerbate genomic instability through ROS/RNS release and cytokine production, creating a tumor-promoting microenvironment. Additionally, chronic inflammation has been implicated in the development of resistance to chemotherapy and radiotherapy by modulating DNA damage response pathways. Understanding the interplay between inflammation, genomic instability, and therapy resistance provides a framework for novel treatment strategies. Targeting chronic inflammation with non-steroidal anti-inflammatory drugs (NSAIDs), corticosteroids, or biological agents such as monoclonal antibodies offers promising avenues for cancer prevention and treatment. Targeting inflammation with NSAIDs, corticosteroids, and monoclonal antibodies shows promise in cancer prevention and therapy, particularly in lung and pancreatic cancer. These agents act by blocking key inflammatory pathways like COX-2, NF-κB, and cytokine signaling. However, potential adverse effects require further clinical evaluation.

## 1. Introduction

Inflammation is a fundamental physiological response to tissue damage resulting from physical injury, trauma, microbial infection, or chemical exposure. Its primary function is to eliminate harmful agents and initiate tissue repair processes [1]. During this response, immune cells—including macrophages—recognize pathogen-associated molecular patterns (PAMPs), leading to the activation of intracellular signaling cascades and the release of pro-inflammatory cytokines, chemokines, and growth factors. These mediators facilitate the neutralization of noxious stimuli, promote apoptosis in compromised cells, and enhance their clearance by phagocytes.

**Figure 1:**
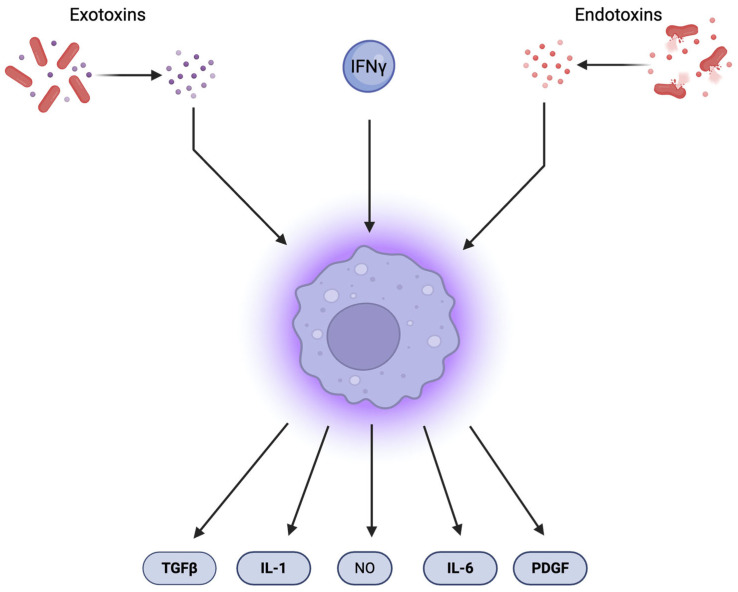
Macrophages are activated by interferon-γ (INF-γ), exotoxins, and endotoxins. Upon activation, they release nitric oxide (NO), tissue-regenerating factors such as platelet-derived growth factor (PDGF), transforming growth factor beta (TGF-β), and cytokines including interleukin 1 (IL-1) and IL-6.

Inflammation can be classified into two types based on duration: acute, which typically resolves within days to weeks, and chronic, which may persist for months or even years. Acute inflammation is characterized by rapid infiltration of neutrophils and macrophages at the site of injury or infection, enabling pathogen elimination and restoration of homeostasis [2]. In contrast, chronic inflammation often fails to resolve the causative factor and involves sustained infiltration by lymphocytes and macrophages. Activated macrophages—stimulated by interferon-γ (IFN-γ), bacterial exotoxins, or endotoxins—produce a wide range of bioactive compounds, including reactive oxygen and nitrogen species, proteolytic enzymes, nitric oxide (NO), eicosanoids, pro-coagulant factors, and cytokines such as IL-1, IL-6, TNF, IL-5, IL-10, and IL-12 [3]. The activation pathways of macrophages and their subsequent release of inflammatory and tissue-regenerating mediators are illustrated in Figure 1. They also release angiogenic factors and collagenases, which contribute to tissue remodeling.

Although inflammation is critical for pathogen defense and wound healing, excessive or unresolved inflammatory responses can be detrimental. Chronic inflammation may lead to tissue destruction, fibrosis, and loss of organ function. Importantly, it is also implicated in the initiation and progression of various cancers. Both exogenous and endogenous factors can drive chronic inflammation. Persistent bacterial and viral infections—such as those caused by hepatitis B and C viruses (HBV, HCV), *Helicobacter pylori*, and human papillomavirus (HPV)—are associated with increased risk of hepatocellular carcinoma, gastric cancer, MALT lymphoma, and cervical, head, and neck cancers, respectively [4]. Parasitic infections, including *Schistosoma* spp., have been linked to bladder cancer, while microbiota imbalances (e.g., involving *Bacteroides* spp.) may contribute to colorectal carcinogenesis. Inhalation of asbestos fibers and exposure to tobacco smoke represent additional environmental risk factors for inflammation-driven lung cancer [5].

Dysregulated immune responses also play a critical role in autoimmune and systemic inflammatory conditions. In diseases such as rheumatoid arthritis, immune cells erroneously target self-tissues, triggering chronic joint inflammation. Moreover, inflammation is implicated in neurodegenerative and cardiovascular disorders. In older individuals, chronic immune activation may contribute to diseases such as Parkinson’s disease, atherosclerosis, myocardial infarction, and heart failure [6]. Obesity is another key contributor to low-grade chronic inflammation, which can worsen cancer outcomes and heighten cancer risk.

Exposure to specific environmental factors, together with the presence of defined genetic determinants, has been associated with an elevated risk of developing inflammation and tumorigenesis. According to research, reducing the intake of saturated fatty acids in the diet may significantly lower the risk of developing various diseases, including cancer [7]. Furthermore, an inadequate diet may contribute to the development of obesity, which is one of the key factors of inflammation and can elevate the risk of breast cancer [8]. Moreover, not only chronic inflammation associated with infections contributes to carcinogenesis, but microorganisms such as bacteria, fungi, and viruses themselves are also strongly implicated as etiological agents in the development of cancer [9].

Notably, inflammation is not only a predisposing factor for malignancy but may also arise as a consequence of anticancer therapies. Treatments such as chemotherapy and radiotherapy can promote tissue damage and immunosuppression, creating a pro-tumorigenic environment that facilitates cancer progression and therapeutic resistance.

## 2. Inflammation-Induced DNA Damage Mechanisms

This section outlines the principal mechanisms linking inflammation to DNA damage, focusing on oxidative and nitrosative stress, direct modifications to nucleic acids, and damage mediated by activated immune cells.

### 2.1. Generation and Physiological Role of Reactive Species

Numerous mediators are involved in the inflammatory response, including reactive oxygen and nitrogen species (ROS and RNS), which comprise a wide spectrum of reactive anionic and neutral molecules generated by various cell types throughout the human body [10,11,12]. While ROS and RNS are primarily byproducts of mitochondrial respiration, a significant additional source is nicotinamide adenine dinucleotide phosphate (NADPH) oxidase, which exists in seven catalytic isoforms differing in tissue localization, activation mechanisms, and roles in pathophysiology [13].

The major reactive species include superoxide anion (O_2_^−^), hydrogen peroxide (H_2_O_2_), hydroxyl radical (HO∙), and nitric oxide (NO) [14]. Although historically considered merely cytotoxic, growing evidence highlights their physiological roles in the redox regulation of protein phosphorylation, ion channel activity, transcriptional control, and in biosynthetic pathways such as thyroid hormone synthesis and extracellular matrix remodeling. Nevertheless, excessive accumulation of ROS leads to oxidative damage to proteins, lipids, and nucleic acids [15].

Oxidative stress, defined as an imbalance between ROS/RNS production and antioxidant defenses, has been implicated in the pathogenesis of neurodegenerative diseases such as Alzheimer’s and Parkinson’s, as well as cardiovascular disorders [15,16]. In the context of cancer, chronic inflammation promotes tumor progression by enhancing cell proliferation, angiogenesis, metastasis, and resistance to therapy [17,18]. The following paragraphs will discuss modifications that lead to gene damage. Damage can affect genes responsible for DNA repair, genes regulating apoptosis, as well as proto-oncogenes and suppressor genes. DNA damage leads to abnormal protein synthesis and disruption of signaling pathways [19]. Malfunctioning DNA repair mechanisms and inhibition of the apoptosis pathway result in the accumulation of cells with damaged genetic material and the development of genomic instability in these cells. The acquisition of a cancerous nature by cells is also contributed to by mechanisms that silence suppressor genes and amplify proto-oncogenes called oncogenes. The resulting cancer cells secrete mediators that modify the tumor microenvironment. As a result, cancer cells are less effectively fought by the immune system. In addition, disruption of signaling pathways caused by DNA damage is the basis for the development of cancer resistance to therapy [20]. The mechanisms presented above are described in detail below.

### 2.2. Oxidative and Nitrosative Modifications of Nucleic Acids

Reactive species are highly reactive due to unpaired electrons, and their accumulation leads to free radical-mediated chain reactions, damaging cellular macromolecules, including DNA [21,22]. Direct mechanisms of nucleic acid damage include nitrosative deamination, oxidation, and halogenation, while indirect mechanisms involve DNA adduct formation with electrophilic products from lipid peroxidation and other metabolic processes [17,23].

A prominent example of DNA oxidation is the modification of guanine at the C8 position, resulting in 8-hydroxyguanine (8-oxoG), a commonly used biomarker of oxidative stress [24,25,26]. Cells counteract this through specific repair enzymes such as 8-oxoguanine DNA glycosylases (OGG), which excise the modified base and maintain genomic stability [26,27].

**Figure 2:**
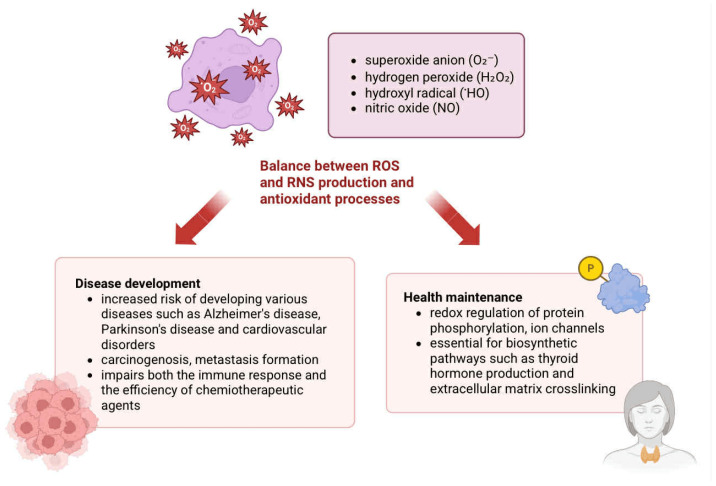
Schematic representation of the potential consequences arising from an imbalance between the production of reactive oxygen species (ROS) and reactive nitrogen species (RNS) and the efficiency of antioxidant defense mechanisms. The generation of key reactive species—including superoxide anion (O_2_•^−^), hydrogen peroxide (H_2_O_2_), hydroxyl radical (•OH), and nitric oxide (NO)—is essential for maintaining physiological homeostasis. As illustrated, these molecules play critical roles in redox signaling, immune modulation, and cellular metabolism, but their excessive accumulation contributes to oxidative stress and subsequent cellular damage.

Nitrosative deamination affects both DNA and RNA and involves hydrolysis, enzymatic activity, and nitrosative reactions [28,29]. Nitric oxide (NO), though essential for physiological processes, can be transformed into nitrous anhydride (N_2_O_3_), which subsequently nitrosates amines and other nucleophiles in DNA [28]. This leads to base deamination, resulting in uracil, hypoxanthine, xanthine, and thymine from cytosine, adenine, guanine, and 5-methylcytosine, respectively [28,30]. Such base modifications may lead to mutagenesis during DNA replication or due to ineffective repair mechanisms [28]. The physiological and pathological roles of ROS and RNS in the context of inflammation-induced cellular damage are visually summarized in Figure 2.

### 2.3. Immune-Mediated Induction of DNA Damage

DNA double-strand breaks (DSBs) represent a severe form of ROS-induced genomic damage and are characterized by phosphorylation of the histone variant H2A.X. This modification is essential for the activation of ATM-mediated DNA damage checkpoints and for the initiation of DSB repair processes [16].

**Figure 3:**
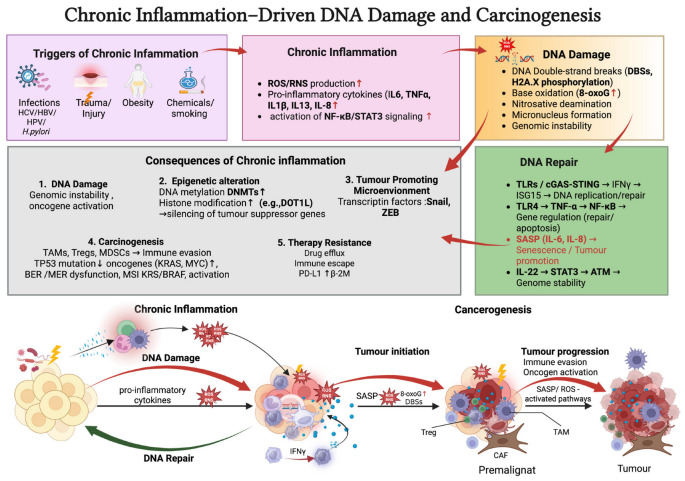
The schematic summarizes the multifaceted relationship between chronic inflammation, DNA damage, and carcinogenesis. Persistent external or internal triggers (light pink box)—such as viral infections (e.g., HCV, HBV, HPV), trauma, obesity, and exposure to chemicals or smoking—initiate chronic inflammation (pink box). This leads to increased production of reactive oxygen and nitrogen species (ROS/RNS, red star icon), pro-inflammatory cytokines (e.g., IL-6, TNF-α, IL-1β, IL-13, IL-8, blue dots), and activation of key signaling pathways (NF-κB, STAT3, red upward arrows, pink box). These inflammatory mediators contribute to DNA damage. (orange box), including double-strand breaks, base oxidation (e.g., 8-oxoG), nitrosative deamination, and micronucleus formation, resulting in genomic instability. In parallel, DNA repair processes (green box) are influenced by pathways such as TLRs, cGAS-STING, and IL-22/STAT3, which may either restore genome integrity or, if dysregulated, promote senescence and tumor development. Downstream consequences include epigenetic alterations, immune evasion, tumor-promoting microenvironments, and therapy resistance, collectively facilitating carcinogenesis.

Recent studies have demonstrated that effector memory CD4^+^ T cells can induce DSBs in dendritic cells via mitochondrial ROS. This activates a cGAS-independent STING–TRAF6–NF-κB signaling cascade, ultimately promoting the expression of inflammatory cytokines such as IL-1β and IL-6 [30]. Furthermore, interleukin-13 (IL-13), commonly secreted in chronic inflammatory environments, has been shown to induce DNA damage manifested by micronucleus formation and protein nitration [31]. Table 1 provides a summary of the key oxidative and nitrosative DNA lesions induced during chronic inflammation, along with their potential mutagenic outcomes and implications for genome stability. An integrated overview of the mechanisms linking chronic inflammation to DNA damage, genomic instability, and cancer progression is presented in Figure 3.

## 3. Influence of Inflammatory Signaling Pathways on DNA Repair

**Figure 4:**
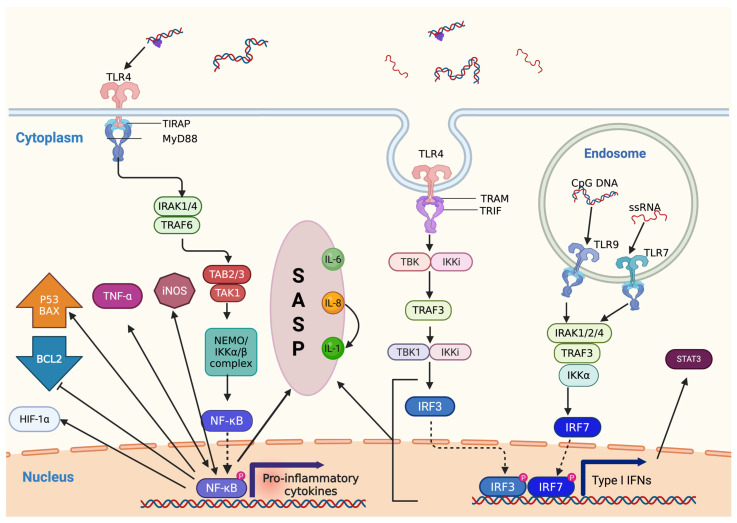
Schematic representation of the signaling cascade following detection of extracellular DNA. Activation of toll-like receptor 4 (TLR4 pink receptor) induces nuclear factor kappa-light-chain-enhancer of activated B cells (NF-κB, violet blue) via tumor necrosis factor alpha (TNF-α, fandango). NF-κB activation leads to upregulation of pro-apoptotic genes Bcl-2-associated protein X (BAX) and tumor protein p53 (TP53) (orange upward arrow), alongside downregulation of the anti-apoptotic B-cell lymphoma 2 (BCL2) gene (cyan blue downward arrow). Additionally, NF-κB enhances hypoxia-inducible factor 1 alpha (HIF-1α -light blue) expression and stimulates inducible nitric oxide synthase (iNOS—dusty red) to produce nitric oxide (NO), which forms a positive feedback loop to NF-κB. NF-κB also regulates the transcription of the senescence-associated secretory phenotype (SASP—dust pink oval), a collection of cytokines, growth factors, and chemokines, including IL-1, IL-6, and IL-8 (CXCL-8). Activation of toll-like receptors 7 (TLR7—blue) and 9 (TLR9—light blue) stimulates interferon type I (INF) expression, which subsequently activates signal transducer and activator of transcription 3 (STAT3, dark purple) and induces IL-6 expression. Arrows denote activation, and blunt ends denote inhibition.

Inflammation plays a pivotal role in modulating the DNA damage response (DDR) and the efficiency of DNA repair mechanisms. This section is divided into three subsections that explore these interactions in detail. An overview of key inflammatory signaling cascades and associated molecules modulating various stages of the DDR is presented in Table 2, while the signaling cascade triggered by extracellular DNA detection and its downstream effects are illustrated in Figure 4.

### 3.1. Inflammatory Mediators and the Modulation of DNA Damage Response

The DNA damage response (DDR) encompasses a sequence of biochemical events initiated upon DNA damage, including damage detection, cell cycle arrest, activation of repair mechanisms, or apoptosis [46,47,48]. Numerous studies have demonstrated that inflammatory mediators—including cytokines (TNF-α, IL-1, IL-6), vasoactive amines (histamine, serotonin), chemokines (e.g., IL-8, MCP-1), and eicosanoids (prostaglandins, leukotrienes)—significantly influence the DDR [49,50]. These molecules not only contribute to DNA damage induction but also modulate the cellular response to it.

Inflammatory signaling pathways become activated shortly after DNA injury. Toll-like receptors (TLRs), such as TLR4, TLR7, and TLR9, along with the cGAS-STING pathway, recognize extracellular DNA [43,45]. For instance, activation of TLR7, TLR9, and cGAS induces type I interferon (IFN) synthesis. IFN, in turn, contributes to tumor suppression by upregulating IL-6 and activating signal transducer and activator of transcription 3 (STAT3). Additionally, IFN enhances the expression of interferon-stimulated gene 15 (ISG15), which accelerates replication fork progression in damaged DNA. Recent findings suggest that ISG15 may protect BRCA-deficient breast cancer cells by stabilizing replication forks [39,40].

### 3.2. Activation of NF-κB Signaling and Its Multifaceted Role in DDR

TLR4 activation induces TNF-α production, which subsequently triggers NF-κB activation and its nuclear translocation. This pathway is reinforced by a positive feedback loop, as NF-κB also enhances TNF-α gene expression [32,33]. In addition, NF-κB may be activated via alternative signaling cascades. For instance, thymidylate kinase (TM) can activate NF-κB by phosphorylating the NF-κB essential modulator (NEMO), which in turn activates IκB kinases (IKKα and IKKβ). The degradation of IκB allows NF-κB to translocate into the nucleus. Moreover, cGAS-mediated synthesis of cyclic GMP-AMP (cGAMP) activates STING, leading to the activation of both IRF3 and NF-κB pathways [44].

NF-κB can also be activated independently of canonical DDR signaling, notably through stress-responsive pathways involving p38MAPKα or the transcription factor GATA4 [34]. Within the DDR context, NF-κB regulates the transcription of a broad spectrum of target genes, including TNF-α, inducible nitric oxide synthase (iNOS), hypoxia-inducible factor 1-alpha (HIF-1α), and components of the senescence-associated secretory phenotype (SASP). It also influences apoptotic responses by upregulating pro-apoptotic genes (e.g., BAX, p53) and repressing anti-apoptotic factors such as BCL2 [34,35,36,37,38,51].

iNOS activation contributes to oxidative stress, establishing a second positive feedback loop that further amplifies NF-κB activity and promotes cytochrome c release. HIF-1α supports metabolic adaptation, enhancing the survival capacity of DNA-damaged cells under hypoxic conditions [37].

### 3.3. Senescence-Associated Secretory Phenotype and Cytokine Feedback Loops

The senescence-associated secretory phenotype (SASP) comprises a wide range of secreted molecules, including cytokines (e.g., IL-1, IL-6), chemokines, and growth factors such as CXCL-8 (IL-8) [41]. While SASP can reinforce cell cycle arrest and limit proliferation in cells with moderate DNA damage, in the context of extensive genomic instability, it may paradoxically support tumorigenic processes [41].

Among SASP factors, IL-6 and IL-8 are particularly pivotal. IL-6 mediates cell cycle arrest by signaling through its receptor and inducing p15INK4B expression, while simultaneously upregulating transcription factors such as PAI-1 and C/EBP, which suppress mitogenic signaling and enhance oxidative stress [41]. IL-8, through CXCR2 receptor activation, also contributes to growth arrest in senescent cells [41].

IL-6 and IL-8 additionally sustain and amplify SASP signaling cascades initially triggered by IL-1, which activates C/EBPβ and NF-κB, further promoting the expression of SASP components [41]. Another cytokine implicated in the regulation of DDR is IL-22, which modulates ataxia telangiectasia mutated (ATM) expression via STAT3, thereby supporting genome stability and limiting the accumulation of mutations [42].

## 4. Role of Immune Cells in Promoting Tumorigenesis

**Figure 5:**
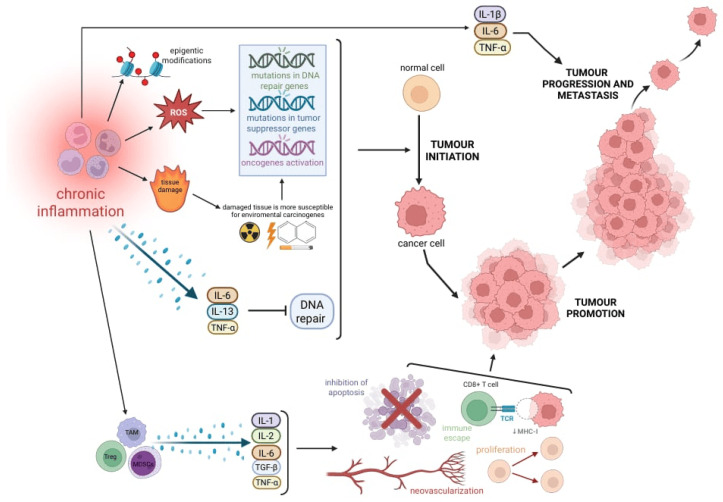
The schematic illustrates the role of chronic inflammation in driving tumorigenesis and cancer progression. A sustained inflammatory microenvironment promotes the generation of reactive oxygen species (ROS), induces epigenetic alterations, and causes tissue injury. These processes collectively increase the likelihood of mutations in DNA repair genes, tumor suppressor genes, and oncogenes, leading to genomic instability and tumor initiation. Pro-inflammatory cytokines, such as IL-6, IL-13, and TNF-α, suppress DNA repair pathways (blunt end), thereby facilitating mutagenesis. Concurrently, tumor-associated macrophages (TAMs—light blue), regulatory T cells (Tregs—green), and myeloid-derived suppressor cells (MDSCs—purple) secrete immunosuppressive and pro-tumorigenic cytokines, including IL-1, IL-2, IL-6, TGF-β, and TNF-α. These factors support cancer cell proliferation, inhibit apoptosis, enable immune evasion, and promote neovascularization. Persistent cytokine signaling and immunosuppressive activity collectively foster tumor promotion, progression, and metastasis.

Chronic inflammation orchestrates multiple stages of tumor development, including initiation, promotion, invasion, and metastasis. Inflammatory cells contribute to these processes through the induction of DNA damage, suppression of DNA repair mechanisms, activation of oncogenic signaling, and remodeling of the tumor microenvironment [17,52,53]. The inflammation-driven mechanisms contributing to tumor initiation and progression are illustrated in Figure 5.

### 4.1. Inflammation-Induced Genomic Instability and DNA Repair Suppression

The chronic inflammatory microenvironment plays a critical role in various stages of tumor development, including initiation, promotion, and invasion [17,52,53]. Inflammatory cells drive DNA damage, mutations, and epigenetic modifications, thereby contributing to genomic instability—a recognized hallmark of tumorigenesis, often detectable even in precancerous lesions [17,52]. Genomic instability is referred to as a state of accumulation of mutations within the genome that changes the activity of plenty of proteins, ultimately resulting in the development of cancer. Furthermore, chronic inflammation provides numerous molecules, facilitating tumor growth, angiogenesis, escape from host immune defense mechanisms, and remodeling of the extracellular matrix (ECM) [54,55,56,57,58].

A prolonged inflammatory milieu creates a permissive environment for tumor initiation. As mentioned previously, the persistent presence of inflammatory cells contributes to DNA damage both directly, through the action of ROS and RNS via oxidation, nitration, and halogenation, and indirectly through their byproducts. Secreted by macrophages and neutrophils, these reactive species induce various types of genomic alterations, including base pair substitutions, small deletions, strand breaks, and cross-linking [17,52,53,59,60,61,62,63]. If not efficiently repaired, these DNA lesions can lead to the dysregulation of key molecular pathways, particularly those involved in cell proliferation, cell cycle checkpoints, and apoptosis—processes critical for carcinogenesis. Indeed, inactivating mutations in tumor suppressor genes (such as TP53) and retinoblastoma transcriptional corepressor 1 (RB1) or activating alterations in oncogenes (e.g., Kirsten rat sarcoma virus (KRAS), myelocytomatosis oncogene (MYC)) are key events in tumor initiation [57,63,64].

These concepts are well supported in research. Notably, Lin et al. demonstrated a distinct correlation between chronic inflammation, DNA damage, and gastrointestinal tumor development. In one study, the research group observed that dysplasia and precancerous lesions were most commonly found in gastric cardia tissues exhibiting high levels of phosphorylated histone H2AX (γH2AX), a reliable marker for DNA DSBs. Importantly, the strongest γH2AX immunostaining in epithelial cells was observed under conditions of severe chronic inflammation, compared to normal gastric cardia tissues [61]. Similar findings were reported in a subsequent study, in which esophageal tissues with higher degrees of chronic inflammation exhibited more intense immunostaining for 8-hydroxy-2′-deoxyguanosine (8-OHdG) and γH2AX. Notably, these samples also displayed dysplastic structures, supporting a positive correlation between chronic inflammation and carcinogenesis [61].

Beyond inducing DNA damage, chronic inflammation also influences tumor development by disrupting DNA repair mechanisms, such as base excision repair (BER) and mismatch repair (MMR). Chronic inflammatory environments can lead to the suppression of DNA repair genes (MutL homolog 1 (MLH1), MutL homolog 2 (MSH2), MutL homolog 6 (MSH6)) through direct mutations induced by ROS and RNS, epigenetic silencing, or cytokine-mediated repression (e.g., TNF-α, IL-6, IL-13) [52,53,65,66,67]. The inactivation of MMR genes results in the accumulation of errors within microsatellite regions—short DNA repeats scattered throughout the genome. This phenomenon, known as microsatellite instability (MSI), has been strongly linked to the accumulation of mutations in oncogenic drivers such as KRAS, neuroblastoma RAS viral oncogene (NRAS), and v-Raf murine sarcoma viral oncogene homolog B1 (BRAF), ultimately contributing to colorectal cancer development [68,69,70]. Furthermore, MSI has been identified in other malignancies, including gastric, endometrial, and bladder cancers [59,68,69,71,72].

### 4.2. IRAK-1 and NF-κB Signaling in Immune-Mediated Tumor Promotion

A growing body of research indicates that interleukin-1 receptor-associated kinase 1 (IRAK-1) signaling may play a significant role in the development and progression of cancer. *Helicobacter pylori*, a bacterium closely linked to gastric inflammation and the onset of gastric cancer, has been shown to cause an upregulation of TLR2 and TLR5 expression across various cell types. This subsequent engagement of these receptors leads to an increase in IRAK-1 phosphorylation and the activation of NF-κB. NF-κB plays a crucial role in regulating the expression of genes that are essential for various cellular processes, including growth, proliferation, anti-apoptosis, angiogenesis, tissue invasion, and metastasis.

Typically, NF-κB fosters cell growth and proliferation by enhancing the expression of cellular myelocytomatosis oncogene (c-myc) as well as cyclin proteins such as D1, D2, D3, E, and cyclin-dependent kinase 2 (CDK2), all of which are key regulators of the cell cycle progression. Additionally, NF-κB promotes cellular growth by producing several growth factors, including IL-2, IL-6, granulocyte macrophage colony-stimulating factor (GM-CSF), and CD40L. Furthermore, NF-κB contributes to the inhibition of apoptosis by regulating anti-apoptotic proteins such as cellular inhibitor of apoptosis proteins (cIAPs), cellular FLICE-like inhibitory proteins (c-FLIP), and various members of the Bcl-2 family. Notably, the activation of NF-κB also enhances angiogenesis within tumors [54,62,63].

Additional evidence highlighting the significance of IRAK-1 in cancer comes from studies focusing on microRNAs (miRNAs). These small non-coding RNA sequences play crucial roles in regulating cellular mRNA stability, protein expression, proliferation, apoptosis, and cancer metastasis. Notably, research has indicated that the expression of a specific miRNA, miR-146a, is often reduced in metastatic prostate cancers [60].

### 4.3. Epigenetic Modifications, EMT, and Pro-Tumor Immune Cells

Additionally, chronic inflammation can promote tumorigenesis through epigenetic modifications. Inflammatory cytokines (e.g., IL-6, TNF-α, IL-1β) can alter the activity of DNA methyltransferases (DNMTs) and histone-modifying enzymes (e.g., disruptor of telomeric silencing 1-like, DOT1L), leading to aberrant epigenetic modifications [5,66,73]. Notably, hypermethylation of tumor suppressor genes can contribute to tumorigenesis by silencing critical regulatory pathways. Collectively, these mechanisms highlight the profound impact of inflammation-induced genomic instability in shaping the evolutionary trajectory of cancer [54,55,64].

Finally, Greten et al. proposed that chronic inflammation may contribute to tumorigenesis by causing tissue damage and exposing cells to environmental carcinogens, further amplifying the processes described above [5].

“Tumor promotion” is a broad concept that encompasses the progression of a tumor from a single transformed cell to a growing mass through the expansion of clonal cells, the formation of new blood vessels, and immune evasion [5,66]. In a prolonged inflammatory environment, a substantial number of immune cells actively contribute to tumor promotion by secreting various signaling molecules. Among them, tumor-associated macrophages (TAMs), myeloid-derived suppressor cells (MDSCs), and regulatory T cells (Tregs) play key roles in shaping the tumor-favorable microenvironment. Several inflammatory mediators, including IL-1, IL-6, IL-23, TGF-β, and TNF-α, have been implicated in this process, primarily through activation of the NF-κB and STAT3 pathways. These signaling cascades promote cell survival, proliferation, angiogenesis, and resistance to apoptosis by upregulating the expression of anti-apoptotic genes (Bcl-xL, Bcl-2, c-IAP2, myeloid cell leukemia-1 (MCL1)) and cell cycle regulators (cyclin D1) [5,65,66,74,75,76,77,78].

Furthermore, chronic inflammation stimulates epithelial–mesenchymal transition (EMT), which is a key process during cancer progression and metastasis. EMT results in the reduction in cellular polarity and intercellular adhesions to form a loosely organized cell structure, which provides motility and invasive abilities for tumor cells. It is favorable for the invasion–metastasis cascade at the primary site, intravasation into blood vessels, and translation to distant tissues [79,80]. The chronic inflammatory environment activates various transcription factors, such as Snail family transcriptional repressors (Snail), Zinc finger E-box-binding homeobox proteins (ZEB), and basic helix–loop–helix factors, through the release of multiple cytokines, including IL-1β, IL-6, TNF-α, and TGF-β, as demonstrated in numerous studies [81,82,83]. Furthermore, TAMs and neutrophils present in a chronic inflammatory milieu secrete matrix metalloproteinases (MMPs) that facilitate tumor invasion through degradation of the ECM [65,66].

Most research indicates a positive link between chronic inflammation and the development of cancer. However, this pattern may be influenced by publication bias, since studies reporting neutral or negative results are less frequently published. Thus, although current evidence strongly suggests that inflammation promotes tumor growth, further investigations are needed to provide a more balanced perspective and address potential biases.

## 5. Therapeutic Strategies to Target Chronic Inflammation in Cancer

**Figure 6:**
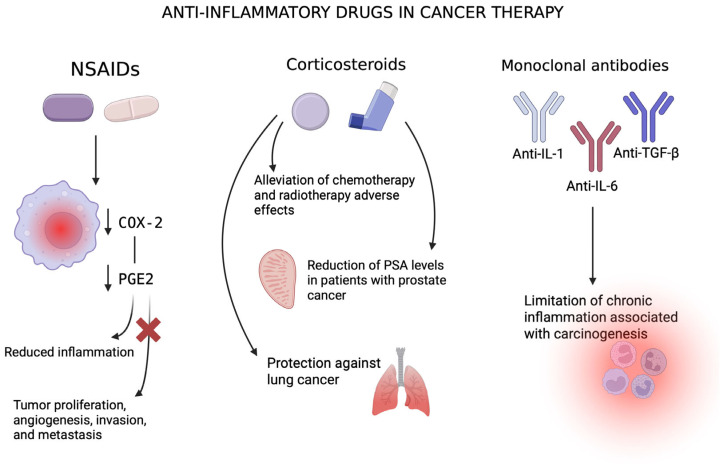
A schematic representation of pharmacological strategies targeting chronic inflammation in cancer therapy and prevention, highlighting their mechanisms of action. The illustration includes non-steroidal anti-inflammatory drugs (NSAIDs), corticosteroids, and monoclonal antibodies that modulate key inflammatory signaling pathways, particularly those involving IL-1, IL-6, and TGF-β. COX-2—cyclo-oxygenase 2, PGE2—prostaglandin E2, PSA—prostate-specific antigen, anti-IL-1—antibody against interleukin-1, anti-IL-6—antibody against interleukin-6, anti-TGF-β—antibody against transforming growth factor beta.

Inflammation plays a fundamental role in carcinogenesis by promoting cellular proliferation and angiogenesis, facilitating metastasis formation, and impairing both the immune response and the efficiency of chemotherapeutic agents. Therefore, targeting inflammatory pathways and their mediators has emerged as a promising strategy in cancer prevention and therapy [18,84]. Various classes of anti-inflammatory agents, including non-steroidal anti-inflammatory drugs (NSAIDs), corticosteroids, and monoclonal antibodies, are under investigation for their potential to mitigate inflammation-associated tumorigenesis. Despite encouraging findings, their clinical utility remains complex and context-dependent. The pharmacological strategies targeting chronic inflammation in cancer therapy and prevention are illustrated in Figure 6.

### 5.1. Non-Steroidal Anti-Inflammatory Drugs (NSAIDs)

NSAIDs exhibit anticancer potential by inhibiting prostaglandin biosynthesis through the blockade of cyclooxygenase (COX) enzymes, particularly COX-2, which is often overexpressed in tumor cells [84,85]. COX-2 and its downstream product prostaglandin E2 (PGE2) participate in critical oncogenic processes such as proliferation, angiogenesis, immune evasion, and metastasis. They exert a direct influence on immune cell function, playing a pivotal role in tumor immune evasion and the establishment of a pro-tumor inflammatory microenvironment. Targeting the COX-2/PGE2 pathway may enhance the efficacy of immune checkpoint blockade (ICB) therapies, which aim to counteract immunosuppressive signals and improve anti-tumor immune responses. Preclinical studies demonstrate that NSAIDs can modify the tumor microenvironment, increase the effectiveness of ICB, and induce a phenotypic shift in T lymphocytes toward an effector state. These findings suggest that anti-inflammatory agents enhance immune surveillance and hinder tumor cells from escaping immune control, highlighting their potential as an effective component of cancer therapy [86,87].

Furthermore, NSAIDs may modulate autophagy, a form of programmed cell death, involved in multiple processes that directly contribute to tumorigenesis. They influence signaling pathways such as PI3K/Akt/mTOR, MAPK/ERK1/2, P53/DRAM, AMPK/mTOR, Bip/GRP78, CHOP/GADD153, and HGF/MET, and inhibit lysosomal function, leading to p53-dependent cell cycle arrest in the G1 phase. NSAIDs have been shown to both activate and inhibit autophagy, with their effects varying depending on the specific tumor context. The precise mechanisms underlying their activity require further investigation [88]. Aspirin, in particular, has been extensively studied in the context of cancer prevention, especially for gastrointestinal malignancies. Long-term aspirin use correlates with reduced incidence, metastasis, and mortality in colorectal cancer. Its primary mechanism of action is likely linked to prostaglandin-related pathways. Furthermore, a correlation has been noted between the duration of aspirin supplementation and its impact on cancer development—its preventive effects appear to increase with prolonged treatment [18,85,89]. In individuals with a history of colorectal adenomas, aspirin reduced the formation of new lesions. Sulindac, another NSAID, has shown beneficial effects in familial adenomatous polyposis [18,90]. Aspirin may also play a protective role in breast cancer development [18,85].

However, the use of NSAIDs is not without risks. Some studies report an association between NSAID use and increased incidence of non-Hodgkin lymphoma and pancreatic cancer [85]. Adverse effects, including gastrointestinal bleeding, ulceration, renal dysfunction, and cardiovascular complications, limit their clinical applicability [18,89]. Therefore, while NSAIDs hold promise—particularly as adjuncts in polytherapy with conventional anticancer agents—further investigation is required to delineate their efficacy and safety.

### 5.2. Corticosteroids

Corticosteroids are commonly employed to manage adverse effects of chemotherapy and radiotherapy, yet emerging evidence suggests their potential antitumor properties [18]. In metastatic castration-resistant prostate cancer, corticosteroids have been shown to alleviate tumor-associated symptoms and reduce prostate-specific antigen (PSA) levels [91]. Additionally, corticosteroids may serve as adjuvants to enhance the tolerability of other anticancer agents.

Inhaled corticosteroids have been investigated for lung cancer prevention in patients with chronic obstructive pulmonary disease (COPD), a condition characterized by chronic inflammation. Lung cancer occurs more frequently in these patients, progresses more aggressively, and is associated with a poorer prognosis. COPD-associated inflammation shares molecular features with lung cancer, including activation of NF-κB, PI3K, and Wnt pathways. The Wnt pathway, originally identified from Drosophila and murine models, regulates proliferation and survival; its dysregulation contributes to chronic inflammation and cancer. Meta-analyses suggest a trend toward reduced lung cancer mortality among COPD patients treated with inhaled corticosteroids, especially at higher doses. However, clinical trials remain inconclusive, and corticosteroids showed no efficacy in halting bronchial dysplasia progression [92]. While corticosteroids may counteract pro-tumor inflammation, their immunosuppressive nature raises concerns, especially in the context of immunotherapy, where they may diminish therapeutic efficacy. Therefore, physicians should carefully evaluate the risk–benefit balance before prescribing them to patients. Further research in this area remains essential [86,93].

### 5.3. Monoclonal Antibodies and Anticytokine Therapies

Targeting specific cytokines and inflammatory mediators represents another approach to mitigate tumor-promoting inflammation. Monoclonal antibodies against transforming growth factor-beta (TGF-β), which may acquire pro-tumorigenic functions during cancer progression, are under investigation. The role of TGF-β varies depending on the stage of the disease. In the early stages of tumorigenesis, it suppresses tumor growth and development, whereas in more advanced stages, it acquires pro-tumorigenic properties, driving uncontrolled proliferation, angiogenesis, and metastasis. Aberrant expression of TGF-β has been reported in hepatocellular carcinoma, colorectal cancer, prostate cancer, lung cancer, and breast cancer. Therefore, suppression of TGF-β may potentially enhance the efficacy of anticancer therapies and requires further investigation [94]. Likewise, blockade of the interleukin-1 (IL-1) axis shows promise. Kanakinumab, an IL-1β inhibitor, reduced lung cancer incidence and mortality but increased the risk of severe infections and sepsis. Conversely, Anakinra, targeting IL-1R1, reduced tumor proliferation in multiple myeloma and slowed disease progression. Anti-IL-1 antibodies also appear to be a crucial addition in enhancing the efficacy of chemotherapy in lymphomas [95,96,97].

Monoclonal antibodies against IL-6, a key pro-inflammatory cytokine, have demonstrated inhibitory effects on multiple myeloma cell proliferation and yielded promising outcomes in prostate and ovarian cancers. Inhibition of the CCL2/CCR2 axis, critical for inflammatory cell recruitment, may also reduce tumor-induced immunosuppression and activate anti-tumor immunity. Particularly, benefits may arise from combining it with checkpoint inhibitors [94,95,96,97]. IL-10 is produced by human tumor cells and, in gastric cancer, contributes to the establishment of an immunosuppressive microenvironment that favors tumor growth. Furthermore, IL-10 expression in tumor-infiltrating regulatory T lymphocytes has been shown to facilitate the exhaustion of intratumoral CD8+ T cells. Conversely, some studies suggest that IL-10 may be used as a form of cancer immunotherapy. Therefore, the therapeutic use of anti-IL-10 antibodies requires further investigation and may be highly context-dependent, varying with tumor type [94]. In numerous malignancies, including prostate, ovarian, hepatic, and breast cancers, pathological production of the pro-inflammatory cytokine TNF-α has been observed. TNF-α has also been implicated in the development of resistance to anticancer therapies. Beyond its role in driving tumor cell proliferation, angiogenesis, and metastatic progression, TNF-α additionally enhances TGF-β signaling. Consequently, therapeutic antibodies targeting TNF-α receptors may represent a promising approach in cancer treatment. Such agents have demonstrated inhibitory effects on tumor cell proliferation in ovarian cancer models [94].

### 5.4. Challenges and Future Perspectives

While anti-inflammatory therapies offer significant potential in cancer management, their effectiveness is modulated by various factors, including tumor type, stage, patient immune status, and treatment duration. Current evidence underscores the importance of combination therapies, integrating anti-inflammatory agents with cytotoxic drugs or immunotherapies. Nonetheless, the lack of large-scale clinical trials and definitive data hampers broad clinical implementation. Personalized treatment approaches, guided by inflammatory biomarkers and genetic profiling, may optimize therapeutic outcomes in the future. Table 3 summarizes the main classes of anti-inflammatory agents, their mechanisms of action, cancer types investigated, and notable limitations. Such comparative frameworks may aid in selecting appropriate candidates for individualized treatment plans.

In conclusion, targeting chronic inflammation represents a compelling strategy in cancer prevention and treatment. While NSAIDs, corticosteroids, and monoclonal antibodies offer potential benefits, further research is essential to confirm their efficacy, minimize risks, and define optimal therapeutic regimens tailored to individual patient profiles.

## 6. Impact of Chronic Inflammation on Cancer Therapy Resistance

**Figure 7:**
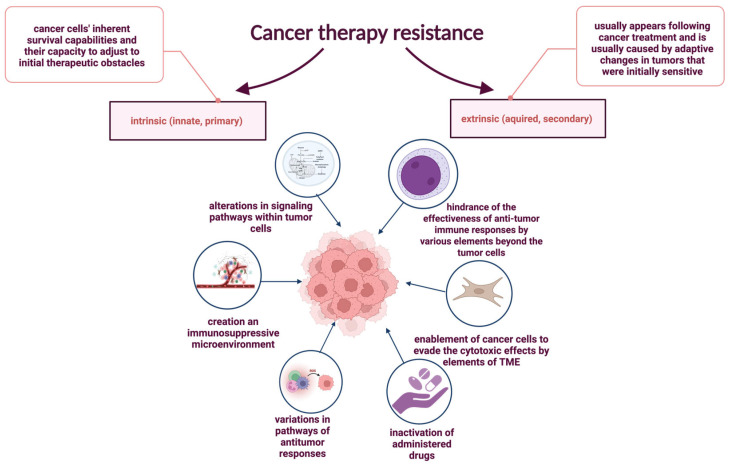
Resistance to anticancer therapy arises through mechanisms influenced by the timing of resistance onset, intrinsic properties of cancer cells, and extrinsic factors within the tumor microenvironment. These mechanisms are categorized as intrinsic and extrinsic resistance. The diagram illustrates the key features and processes involved in each of these resistance pathways.

Therapeutic resistance, common in oncology and other medical fields, refers to reduced treatment efficacy despite using proven therapies [98,99,100,101]. It contributes to about 90% of cancer-related deaths [102]. Cancer cells develop resistance through various mechanisms, including drug inactivation, impaired uptake or increased efflux, apoptosis inhibition, metabolic and epigenetic changes, enhanced DNA repair, and tumor microenvironment influences [98,102,103]. Resistance is classified as intrinsic or acquired. Intrinsic resistance results from pre-existing mutations and tumor heterogeneity [102,104,105], while intrinsic immune resistance also involves altered signaling and immunosuppressive microenvironments [104,106]. The mechanisms underlying resistance to anticancer therapy, including both intrinsic and extrinsic pathways influenced by cellular properties and the tumor microenvironment, are illustrated in Figure 7.

### 6.1. Intrinsic Resistance Mechanisms and Inflammatory Signaling

Intrinsic resistance refers to pre-existing cellular features that enable tumor cells to evade treatment from the outset [98,102,107]. This includes enhanced survival capabilities due to genetic mutations, tumor heterogeneity, and activation of pro-survival signaling pathways [102,104,105]. Chronic inflammation induces epigenetic modifications such as acetylation, sumoylation, phosphorylation, methylation, ubiquitination, and DNA methylation, contributing to gene silencing and resistance [108,109].

One example is the p53 pathway, frequently disrupted in cancers. Loss-of-function mutations in p53 lead to heightened NF-κB activity, which stimulates pro-inflammatory cytokine production and the recruitment of tumor-promoting immune cells such as macrophages [110]. In addition, mutant p53 activates ROS, triggering the JAK-STAT pathway, increasing neutrophil, macrophage, and CD4^+^ T cell infiltration, and suppressing CD8+ T cell responses, weakening antitumor immunity [111,112,113]. The MYC further shapes the tumor’s inflammatory environment. MYC overexpression in transgenic β-cell mouse models induces pro-inflammatory cytokine production (IL-1β, CCL5), promoting angiogenesis and immune cell recruitment. These changes contribute to resistance in both hematological and solid tumors, including leukemia, lymphoma, melanoma, lung, colon, breast, and cervical cancers, by promoting tumor angiogenesis, attracting pro-tumoral mast cells, and driving disease progression through cytokine signaling—mechanisms particularly implicated in acute lymphoblastic leukemia, acute myeloid leukemia, classic Hodgkin lymphoma, multiple myeloma, and are associated with poorer prognosis [111,114,115,116,117,118,119,120,121,122,123].

### 6.2. Extrinsic Resistance and Tumor Microenvironment (TME) Interactions

Extrinsic resistance typically emerges following cancer treatment and is predominantly driven by adaptive changes in initially sensitive tumors. These modifications progressively diminish therapeutic efficacy over time [104]. Extrinsic mechanisms are largely mediated by components of the tumor microenvironment (TME), which actively contribute to cancer cell evasion of various anticancer therapies [124,125]. Key elements of the TME include an altered extracellular matrix (ECM), tumor-associated stromal cells, growth factors, extracellular vesicles (EVs), cancer-associated fibroblasts (CAFs), and diverse immune cell populations. A dense and rigid ECM, commonly observed in tumors, impairs drug penetration and facilitates drug sequestration through direct binding, representing a significant barrier to effective therapy and a critical mechanism of resistance in many solid malignancies [126].

Other tumor-extrinsic mechanisms of adaptive resistance involve immunosuppressive components of the TME, such as regulatory T cells (Tregs), myeloid-derived suppressor cells (MDSCs), and M2-polarized macrophages, all of which can suppress anti-tumor immune responses and compromise the efficacy of immunotherapies [127]. Acquired resistance may also arise via disruption of interferon-gamma (IFN-γ) signaling. A notable example includes loss-of-function mutations in JAK1/2, particularly JAK2, which have been detected in melanoma patients experiencing relapse during anti-PD-1/PD-L1 therapy [128]. Programmed death-ligand 1 (PD-L1), normally expressed by macrophages, activated T and B lymphocytes, dendritic cells, and certain epithelial cells under inflammatory conditions, is also upregulated by tumor cells as an adaptive immune evasion strategy [129]. Furthermore, β2-microglobulin (β2M), an essential component of major histocompatibility complex class I (MHC-I) molecules, is crucial for antigen presentation to CD8+ cytotoxic T lymphocytes [130]. Truncating mutations in the β2M gene can impair antigen presentation, allowing tumor cells to escape immune surveillance. Downregulation or complete loss of β2M expression has been observed in patients with lung cancer and melanoma who initially responded to immune checkpoint inhibitors (ICIs) but subsequently developed resistance [131,132].

### 6.3. Inflammatory Modulation of Drug Metabolism

Inactivation of anticancer drugs represents a major mechanism of therapeutic resistance [98,102]. A key contributor to this process is the cytochrome P450 (CYP) enzyme system, predominantly localized in the liver and intestines. These enzymes are central to the metabolism of various chemotherapeutic agents, including both classical drugs such as doxorubicin and cyclophosphamide, as well as targeted therapies, e.g., imatinib and other tyrosine kinase inhibitors (TKIs) [15,22]. Notably, CYPs are especially critical for the activation of prodrugs, which require enzymatic modification to exert their therapeutic effects [133].

Beyond their detoxifying role in xenobiotic metabolism, CYP enzymes are involved in endogenous processes, such as lipid and fatty acid metabolism, thereby contributing to cellular homeostasis [133]. However, their activity may also promote tumorigenesis. Certain CYP isoforms are capable of bioactivating harmless compounds into carcinogens, inducing aberrant angiogenesis and promoting epigenetic alterations, including methylation of DNA nitrogen bases [133].

In many advanced malignancies, systemic inflammation alters CYP expression and activity, often with significant pharmacokinetic consequences [133,134]. Inflammatory cytokines such as interleukin-1β (IL-1β), interleukin-6 (IL-6), and tumor necrosis factor-alpha (TNF-α) have been shown to downregulate CYP expression, particularly CYP3A4, one of the most clinically relevant isoforms [134,135].

IL-6, for example, activates the PI3K/AKT signaling pathway and modulates CYP gene expression through NF-κB-mediated inhibition of the CYP promoter regions. Additionally, IL-6 can interfere with the pregnane X receptor (PXR) and retinoid X receptor (RXR) dimerization via JAK/STAT and RAS pathways, ultimately reducing transcriptional activation required for CYP mRNA synthesis [134].

### 6.4. Inflammation-Induced Radioresistance

Throughout the course of their illness, nearly half of all cancer patients undergo radiation therapy, which continues to be a critical element of cancer treatment. As a result of its contribution, it has become the primary treatment for cancer for many years [136]. However, it should be noted that cancer cells react to radiotherapy in many ways, which in some instances might contribute to radioresistance. It combines intrinsic determinants along with extrinsic variables. Intrinsic encompasses pathways that favor survival, increased DNA repair ability, protection against oxidative stress, dysfunctional cell cycle arrest, and apoptosis. Additionally, hypoxic microenvironments and protective cellular components like non-CSCs, fibroblasts, immune cells, endothelial cells, soluble factors, and extracellular matrix are incorporated into the extrinsic factors [137,138]. Studies have also shown that proteins facilitating inflammation have been expressed in cancer cells of esophageal adenocarcinomas resistant to radiotherapy, which indicates that inflammation serves a role in regulating the response to ionizing radiation and, as a consequence, indirectly promotes resistance to radiotherapy [139]. Resistance to radiotherapy in breast cancer is also facilitated by the cytokine IL-6, which is recognized for its pro-inflammatory properties and is overexpressed in numerous cancers, especially by adipocytes associated with breast cancer [140]. STAT3 is believed to be a major factor in inflammation since pro-inflammatory cytokines activate that oncogene and can play a major part in resistance against radiotherapy. The STAT3 dimer after activation via cytokines induces other genes, including MCL-1, BCL-xL, and B-cell lymphoma 2 (BCL-2). After exposing ionizing radiation to triple-negative breast cancer cells, STAT3 promotes the expression of a crucial anti-apoptotic gene, BCL-2, which leads to acquired radioresistance [141,142].

### 6.5. Inflammation-Induced Resistance to Immunotherapy

Immunotherapy has emerged as one of the most innovative and effective strategies in modern oncology [143]. In contrast to conventional modalities such as surgery, chemotherapy, and radiotherapy, immunotherapeutic approaches—particularly those utilizing monoclonal antibodies—enable the immune system to recognize and target metastatic tumor cells [143]. A meta-analysis by Ling et al. demonstrated that immunotherapy significantly prolongs overall survival (OS) and progression-free survival (PFS) in cancer patients [143].

These markers can exhibit a direct effect on many aspects, including inflammation. According to the study, patients with metastatic renal cell carcinoma receiving PD-1/PD-L1 inhibitors had a higher baseline neutrophil-to-lymphocyte ratio, an inflammatory prognostic indicator, which was linked to a lower objective response rate (ORR), a shorter progression-free-survival (PFS), and a shorter overall survival (OS) [144]. Additional proof of the influence of inflammation on immunotherapy is seen in its direct effect via JAK-STAT-mediated chronic inflammation, which hinders the activation of cytotoxic T lymphocytes and reduces the effectiveness of anti-PD-1 immunotherapy in pancreatic cancer. This is supported by evidence that the JAK-STAT inhibitor, Ruxolitinib, successfully reduces systemic inflammation within the tumor microenvironment, thereby increasing the infiltration and activation of CTLs and helping to counteract the resistance of pancreatic cancer to anti-PD-1 immunotherapy [145].

However, despite its clinical success, resistance to immunotherapy remains a considerable challenge. Tumor cells can develop or possess mechanisms of immune evasion, reducing the efficacy of immune-based treatments [146]. Notably, chronic inflammation, often associated with tumor progression, induces epigenetic alterations such as DNA methylation and histone modifications in epithelial and cancer cells [130]. These modifications contribute to the transcriptional silencing of genes critical for antigen processing and presentation, including those encoding tumor antigens and major histocompatibility complex (MHC) class I and II molecules [147,148]. Consequently, these changes impair effective tumor recognition by cytotoxic T cells, facilitating immune escape and contributing to acquired resistance [106].

Furthermore, multiple intracellular pathways have been implicated in mediating immunotherapy resistance. Among them are aberrations in the MAPK signaling cascade, loss of PTEN expression resulting in PI3K/AKT pathway hyperactivation, and deficiencies in IFN-γ signaling, which can compromise T-cell-mediated responses [149]. Additionally, activation of the WNT/β-catenin pathway and downregulation or loss of tumor antigen expression have been linked to impaired T-cell infiltration and reduced therapeutic responsiveness [127,149].

## 7. Summary, Future Perspectives, and Alternative Hypotheses

Chronic inflammation contributes to both tissue regeneration and disease development by producing mediators that can damage DNA, including reactive oxygen and nitrogen species, IL-13, and immune cells such as CD4^+^ T lymphocytes. Inflammatory signals trigger DNA repair responses but may also disrupt cell cycle control and promote survival of damaged cells. Prolonged inflammation fosters a tumor-supportive environment, impairs DNA repair, and alters molecular pathways, facilitating carcinogenesis, progression, and metastasis. Understanding these mechanisms is essential for improving cancer prevention and therapy. Future strategies should integrate anti-inflammatory approaches—targeting key pathways and modulating the tumor microenvironment—to overcome therapy resistance and enhance treatment outcomes.

The close link between inflammation and carcinogenesis was presented throughout this research paper. Although to fully grasp the complexity of tumorigenesis, it is essential to explore its other prospective causes. Those might include inherited genetic factors. For instance, the enzyme 5,10-methylenetetrahydrofolate reductase (MTHFR) is crucial for DNA synthesis, methylation, and repair. C677T and A1298C are polymorphic variations in the MTHFR gene. Those variants are proven to decrease the activity of the enzyme, which leads to increased risk of breast cancer and leukemia [150,151].

The possible cancer contributors also include the genotoxic substances and materials such as HEMA and BPA. 2-hydroxyethyl methacrylate (HEMA) is proven to have a pro-apoptotic effect, but is also associated with intracellular ROS accumulation. Breast cancer, prostate cancer, testis cancer, and ovarian cancer are all linked to bisphenol-A (BPA) because of its endocrine-disruptive properties [152,153,154].

Another trigger of tumorigenesis is the change in human DNA caused by biological factors. A well-known example is cervical cancer evoked by the integration of Human Papillomavirus DNA into human DNA. Nevertheless, the relevance between pathogens and carcinogenesis is generally more intricate. The correlation between periodontitis and the increased risk of head and neck cancer is a fair depiction of that concept. The periodontitis-associated microbiome of the oral cavity induces changes in gene expression regulation, such as downregulation of the p53 pathway. The toxins and metabolites released by bacteria contribute to the genetic damage of epithelial cells. However, the altered microbiome also leads to inflammation, hence supporting the model of inflammation-induced cancer [155,156].

Due to the inflammation being the main focus of this work, other aspects of cancer therapy resistance were not wildly explored. Nonetheless this topic is multidimensional, as the number of cancer types and treatment options is vast. In order to provide the solution, other possible causes of therapy resistance must be taken into consideration. For instance, some pre-existing genetic mutations might negate the drug’s effectiveness. Cisplatin is proven to be less efficient in gastric cancer therapy if patients have human epidermal growth factor receptor upregulation. Another reason for developing therapy resistance is tumor heterogeneity. For example, in breast cancer, some neoplastic cells have mutations making them therapy resistant. Under pressure generated by the drug, only the resistant cells will survive, leading to the development of the therapy-resistant population. Different explanations of cancer therapy resistance involve mesenchymal stem cell-derived exosomes. Their ability to modulate apoptosis-related proteins is the cause of 5-FU resistance in gastric cancer cells in the bone marrow microenvironment. The carboplatin resistance of breast cancer is also attributed to MSC-derived exosomes [156,157,158].

The inflammation was proven to be an influential factor in tumorigenesis and therapy resistance, but not the only one. Considering all of the statements above, the open-minded approach is suggested.

## 8. Conclusions

Chronic inflammation plays a central role in both intrinsic and acquired resistance to cancer therapies. It contributes to drug resistance through genetic and epigenetic changes, modulation of the tumor microenvironment, interference with drug metabolism, and suppression of antitumor immune responses.

## Figures and Tables

**Table 1:** Types of inflammation-induced DNA damage, their molecular origin, and biological consequences.

Mechanism	Mediators/Molecules	Type of DNA Damage	Biological Consequences	References
Oxidative damage	ROS: O_2_^−^, H_2_O_2_, HO•	Base oxidation (e.g., 8-oxoG), single- and double-strand breaks (SSBs, DSBs)	Mutagenesis, replication errors, genomic instability	[15,17,21,24]
Nitrosative deamination	NO, N_2_O_3_	Deamination of cytosine, adenine, guanine, 5-methylcytosine → uracil, hypoxanthine, xanthine, thymine	Transition mutations, mispairing, inadequate repair	[28,30]
Lipid peroxidation byproducts	4-HNE, MDA (from PUFA oxidation)	Formation of DNA adducts	Interference with replication and transcription	[23]
Protein nitration and DNA lesions	IL-13, RNS (NO-derived)	Protein nitration, micronucleus formation	Chromosomal aberrations, DNA fragmentation	[31]
Double-strand breaks (DSBs)	ROS, ATM activation, H2A.X phosphorylation	DNA strand scission	Cell cycle arrest, apoptosis, senescence	[16]
Immune-cell-induced damage	Mitochondrial ROS from CD4^+^ T cells; STING–TRAF6–NF-κB axis	Indirect DNA damage via cytokine release (IL-1β, IL-6) and DSBs in dendritic cells	Local immunopathology, activation of inflammatory signaling	[30]

**Table 2:** Key inflammatory pathways and mediators modulating DNA damage response (DDR).

Mediator/Pathway	Mechanism of Action	Effect on DDR and Genome Stability	References
TNF-α	Activates NF-κB via TNFR signaling	Induces transcription of inflammatory genes and oxidative stress responses	[32,33]
NF-κB	Activated by TLRs, thymidylate kinase (TM)/NEMO/IKK, cGAS-STING, p38MAPKα, or GATA4	Regulates expression of iNOS, HIF-1α, SASP components, and apoptotic genes	[34,35,36,37,38]
Interferons (IFNs)	Triggered by TLR7/9 and cGAS-STING, promote STAT3 activation and ISG15 expression	Enhance DNA repair capacity, promote replication fork stability	[39,40]
ISG15	Upregulated by IFN signaling	Supports replication fork stabilization in BRCA-defective breast cancer cells	[40]
IL-6, IL-8 (SASP cytokines)	Maintain senescence signaling via STAT3, CXCR2, and C/EBP transcription factors	Reinforce cell cycle arrest and DNA repair in low-damage conditions	[41]
IL-1	Enhances NF-κB and C/EBPβ activity, sustaining the SASP	Promotes persistent inflammatory signaling and modulates DDR	[41]
IL-22	Activates ATM transcription through STAT3	Prevents accumulation of DNA mutations via enhanced DDR	[42]
cGAS–STING pathway	Detects cytosolic DNA, synthesizes cGAMP, activates IRF3 and NF-κB	Promotes IFN responses and links inflammation with DDR	[43,44]
TLRs (e.g., TLR4, TLR7, TLR9)	Recognize extracellular DNA or pathogens, trigger downstream NF-κB and IFN pathways	Indirectly modulate DDR via inflammatory cytokine production	[43,45]
iNOS (inducible nitric oxide synthase)	Upregulated by NF-κB signaling	Increases nitrosative stress, enhancing DNA damage and DDR activation	[35,37]

**Table 3:** Overview of anti-inflammatory therapeutics in cancer. ↓ indicates a decrease.

Drug Class	Representative Agents	Mechanism of Action	Cancer Types Studied	Limitations
NSAIDs	Aspirin, Sulindac, Celecoxib	COX-2 inhibition, ↓ PGE2, immune modulation	Colorectal, Breast, Pancreatic	GI bleeding, CV risk, renal toxicity
Corticosteroids	Dexamethasone, Prednisone	Broad immunosuppression, NF-κB/PI3K inhibition	Prostate, Lung (COPD-related)	Immunosuppression, ↓ immunotherapy response
Anti-cytokine mAbs	Canakinumab, Anakinra, Anti-IL-6	IL-1β/IL-6/TGF-β blockade, ↓ immune evasion	Lung, Myeloma, Prostate, Ovarian	Infection risk, incomplete clinical validation
Chemokine Inhibitors	Anti-CCL2/CCR2 mAbs	Blocks immune cell recruitment, ↓ TME suppression	Breast, Lung, Pancreatic (experimental)	Early-stage trials, immunologic compensation

## Data Availability

Not applicable.

## Abbreviations

The following abbreviations are used in this manuscript:

8-OHdG	8-hydroxy-2′-deoxyguanosine
8-oxoG	8-hydroxyguanine
AKT	protein kinase B
anti-IL-1	antibody against interleukin-1
anti-IL-1R1	antibody against interleukin-1 receptor type 1
anti-PD1/L1	antibody against programmed death receptor 1/ligand 1
ATM	ataxia-telangiectasia mutated
BAX	Bcl-2-associated protein X
BCL2	B-cell lymphoma 2
Bcl-xL	B-cell lymphoma-extra large
BER	base excision repair
BRAF	B-Raf proto-oncogene, serine/threonine kinase
BRCA	breast cancer
C/EBPβ	CCAAT/enhancer-binding protein beta
CAFs	cancer-associated fibroblasts
CCL2	C-C motif chemokine ligand 2
CCL5	C-C motif chemokine ligand 5
CCR2	C-C motif chemokine receptor 2
CD40L	CD40 ligand
CDK2	cyclin-dependent kinase 2
CEBP	CCAAT/enhancer-binding protein
c-FLIP	cellular FLICE-like inhibitory protein
cGAMP	cyclic guanosine monophosphate-adenosine monophosphate
cGAS	cyclic GMP-AMP synthase
cIAPs	cellular inhibitor of apoptosis proteins
c-myc	cellular myelocytomatosis oncogene
COPD	chronic obstructive pulmonary disease
COX	cyclo-oxygenase
CXCL-8	C-X-C motif chemokine ligand 8
CXCR-2	C-X-C motif chemokine receptor 2
CYP	cytochrome P450
DDR	DNA damage response
DNMTs	DNA methyltransferases
DOT1L	disruptor of telomeric silencing 1-like
DSBs	DNA double-strand breaks
ECM	extracellular matrix
EMT	epithelial–mesenchymal transition
EVs	extracellular vesicles
GATA4	GATA binding protein 4
GM-CSF	granulocyte macrophage colony-stimulating factor
H_2_O_2_	hydrogen peroxide
HBV	hepatitis B virus
HCV	hepatitis C virus
HIF-1α	hypoxia-inducible factor 1-alpha
HO·	hydroxyl radical
HPV	human papillomavirus
ICB	immune checkpoint blockade
ICI	immune checkpoint inhibitor
IKK	IκB kinase
IL-1	Interleukin-1
IL-10	Interleukin-10
IL-12	Interleukin-12
IL-13	Interleukin-13
IL-1β	Interleukin-1 beta
IL-5	Interleukin-5
IL-6	Interleukin-6
IL-8	Interleukin-8
INF-γ	interferon-γ
iNOS	inducible nitric oxide synthase
IRAK-1	interleukin-1 receptor-associated kinase 1
IRF3	interferon regulatory factor 3
ISG15	IFN-stimulated gene 15
JAK	Janus kinase
KRAS	Kirsten rat sarcoma viral oncogene homolog
MALT	mucosa-associated lymphoid tissue
MCL1	myeloid cell leukemia-1
MCP-1	monocyte chemoattractant protein-1
MDSCs	myeloid-derived suppressor cells
MHC-I	major histocompatibility complex class I
miR-146a	microRNA 146a
miRNAs	microRNAs
MLH1	MutL homolog 1
MMPs	matrix metalloproteinase
MMR	mismatch repair
MSH2	MutL homolog 2
MSH6	MutL homolog 6
MSI	microsatellite instability
MYC	myelocytomatosis oncogene
N_2_O_3_	nitrous anhydride
NADPH	nicotinamide adenine dinucleotide phosphate
NEMO	NF-κB essential modulator
NF-κB	nuclear factor kappa-light-chain-enhancer of activated B cells
NO	nitric oxide
NRAS	neuroblastoma RAS viral oncogene homolog
NSAIDs	non-steroidal anti-inflammatory drugs
O_2_^−^	superoxide anion
OGG	8-oxoguanine DNA glycosylases
p15INK4B	cyclin-dependent kinase inhibitor 2B
PAI-1	plasminogen activator inhibitor-1
PDGF	platelet-derived growth factor
PD-L1	programmed death-ligand 1
PGE2	prostaglandin E2
PI3K	phosphatidylinositol 3-kinase
PTEN	Phosphatase and TENsin homolog deleted on chromosome 10
PXR	pregnane X receptor
RAS	rat sarcoma gene
RB1	retinoblastoma transcriptional corepressor 1
RNS	reactive nitrogen species
ROS	reactive oxygen species
RXR	retinoid X receptor
SASP	senescence-associated secretory phenotype
Snail	Snail family transcriptional repressor
STAT3	signal transducer and activator transcription 3
STING	stimulator of interferon genes
TAMs	tumor-associated macrophages
TGF-β	transforming growth factor beta
TKI	tyrosine kinase inhibitor
TLR4	Toll-like receptor 4
TLR5	Toll-like receptor 5
TLR7	Toll-like receptor 7
TLR9	Toll-like receptor 9
TM	thymidylate kinase
TME	tumor microenvironment
TP53	tumor protein p53
TRAF6	tumor necrosis factor receptor-associated factor 6
Tregs	regulatory T cells
Wnt	wingless-type MMTV integration site family
ZEB	zinc finger E-box binding homeobox
β-2M	beta-2-microglobulin
